# Using patient-specific computational fluid dynamics to assess intraprosthetic thrombus formation risk and guide relining after endovascular aneurysm repair

**DOI:** 10.1016/j.jvssci.2026.100437

**Published:** 2026-08-05

**Authors:** Lysette V. Scheepmaker, Paul van Schaik, Lennart van de Velde, Michel M.P.J. Reijnen

**Affiliations:** a Department of Surgery, Rijnstate, Arnhem, The Netherlands; b Multi-Modality Medical Imaging Group, TechMed Centre, University of Twente, Enschede, The Netherlands; c Department of Surgery, Amsterdam UMC, University of Amsterdam, Amsterdam, The Netherlands; d Amsterdam Cardiovascular Sciences, Atherosclerosis and Aortic Diseases, Amsterdam, The Netherlands

**Keywords:** Computational fluid dynamics, Endovascular aneurysm repair, Intraprostethic thrombus formation, Thromboembolic events, Residence time

## Abstract

A patient-specific computational fluid dynamics (CFD) analysis was performed to explore hemodynamic contributors of recurrent intraprosthetic thrombus and limb thrombosis in a patient after endovascular aneurysm repair (EVAR) and to investigate the potential of CFD to guide revision strategies. Key hemodynamic parameters, Time-averaged wall shear stress (TAWSS) and residence time (RT), were assessed showing a region with depressed TAWSS (below 0.1 Pa) at the site of intraprosthetic thrombus and a quasi-infinite RT of entrapped particles in that area. Next, CFD simulations tested a virtual limb-relining configuration, with geometric modifications resulting in improved TAWSS and RT. After mimicking the CFD-based relining with the implantation of an endograft a postprocedural analysis was performed. The postoperative results were in line with the prediction model and showed a 544% reduction in low area of TAWSS (<0.1 Pa) and a normalization of RT to 5 seconds. A contrast-enhanced computed tomography 1 year after stent placement showed a patent endograft without any signs of recurrent mural thrombus. Therefore, CFD may provide valuable insights into the hemodynamic mechanisms underlying endograft thrombosis and may support personalized planning of relining strategies to reduce recurrent thromboembolic risk.

**Clinical relevance:**

The second most common complication after endovascular aneurysm repair is thrombosis in one of the iliac stent graft limbs or distal thromboembolic events, which can cause acute limb ischemia. In some patients, these events persist even after a successful relining of the iliac stent graft with normal flow profiles on ultrasound. This report describes how computational fluid dynamics can identify flow pattern abnormalities and high residence time in a patient with recurring thromboembolic events after endovascular aneurysm repair placement, coinciding with the location of thrombus formation. Moreover, relining strategies can be virtually assessed in the patient's computer model to select a strategy that is expected to normalize the increased residence time.

Limb thrombosis following endovascular aneurysm repair (EVAR) is a relatively common and potentially limb-threatening complication, with an overall incidence exceeding 5%.^1^, ^2^, ^3^ Multiple risk factors contribute to its occurrence, including patient-related factors (eg, nicotine abuse and younger age), anatomical characteristics (eg, increased vessel angulation and tortuosity, smaller iliac artery diameter), stent graft characteristics (eg, geometry, graft material), and procedural aspects (eg, excessive graft oversizing, type of endograft used). Several of these factors influence the postprocedural vascular geometry and may lead to hemodynamic disturbances, such as regions of low wall shear stress (WSS) and prolonged residence time (RT), which in turn may promote thrombus formation.

Computational fluid dynamics (CFD) is a numerical technique used to simulate blood flow dynamics using patient-specific models reconstructed from computed tomography angiography (CTA) data. This type of analysis enables visualization of patient-specific blood flow patterns and the quantification of key hemodynamic parameters, including WSS and RT. CFD has been used for in vitro artificial surfaces to investigate the effect of stent graft-induced step transitions (ie, relatively abrupt increase in lumen diameter at areas of reverse taper) on thrombus formation. The results showed that larger step transitions were associated with increased thrombus formation.^4^ The dynamic formation and shedding of thrombus next to an abrupt reverse taper has also been documented in vitro with magnetic resonance imaging,^5^ and an increased degree of reverse taper has been associated with stent graft thrombosis in sidebranches.^6^ Others studied the relationship between hemodynamic flow parameters and thrombus formation in the Anaconda endograft and suggested that RT, which is related to time-averaged WSS (TAWSS), may be a valuable predictor of limb thrombosis.^7^ Finally, CFD has been applied to predict endograft thrombosis in the fenestrated EVAR sidebranches^8^ and in the superficial femoral artery^9^ by analyzing TAWSS. These studies found that patients who developed thrombosis exhibited regions with unfavorable WSS profiles.^9^

We present the case of a patient with repeated thromboembolic events at the left limb after EVAR that was associated with thrombus in the endograft, without a detected underlying anatomical or hematological cause. CFD analysis revealed a region of disturbed hemodynamics adjacent to an area of reversed tapering of the stent graft. Although it remains uncertain whether this finding is the definitive cause of the recurrent thrombosis, it was the only notable abnormality identified. Based on this observation, a virtual relining of the patient-specific vascular geometry was performed using CFD to assess whether the hemodynamic environment could be favorably modified.

The insights obtained from the CFD simulation were subsequently used to guide the actual relining procedure. This report illustrates both the applied methodology and clinical utility of CFD in supporting treatment decisions for EVAR-related limb occlusion.

## Case description

In 2024, a 73-year-old man was referred as a second opinion because of recurrent thromboembolic events of the left limb after EVAR. The patient had a history of carotid endarterectomy for stroke, hypertension, and a functional solitary kidney with an estimated glomerular filtration rate of 41 mL/min/1.73 m^2^. His current pharmacological regimen consisted of clopidogrel, acenocoumarol, metoprolol, amlodipine, rosuvastin, and ezetimibe. The patient underwent open surgery in 2011 with a tube graft implanted for a ruptured abdominal aortic aneurysm. Later that year, an adjunctive EVAR procedure was performed for an enlarging left-sided common iliac artery aneurysm using an Excluder abdominal aortic aneurysm Endoprothesis main body (W. L. Gore and Associates), with a contralateral gate diameter of 13 mm, and a contralateral Endurant limb (Medtronic) with a distal diameter of 24 mm for the left common iliac (Table).

**Table:** Overview of implanted stent grafts of the left iliac circuit

Years	Stent graft	Diameters, mm	Length, mm
2014	Gore Excluder RLT-26-12-18	Contralateral gate, 13	180
	Medtronic Endurant ETLW-16-16-C82 (bridge) and ETLW-16-24-C82	Proximal, 16; distal, 24	82
2017	Medtronic Endurant ETLW-16-13-C156	Proximal, 16; distal, 13	156
2024	Gore VIABAHN PAHR-13-100-2E	13	100

After this procedure, a total of four thromboembolic events occurred, all on the left side. During the first event in 2015, a thrombectomy of the left EVAR limb and the popliteal artery was performed. Completion angiography revealed embolic occlusions in the ipsilateral internal iliac artery, which were treated with catheter-directed chemical thrombolysis. Postprocedural imaging demonstrated adequate outflow to the lower limb, with patency of both the deep and superficial femoral arteries, as well as all three crural arteries supplying the foot. Following the procedure, a hematology consultation was requested to evaluate for underlying coagulation disorders. A comprehensive analysis was performed, and no significant coagulation disorder was identified.

In 2016, the patient presented with acute ischemia of the left leg, for which a surgical embolectomy was performed of the popliteal and crural arteries complemented by a four-compartment fasciotomy. The procedure was complicated by a rebleed with an associated myocardial infarction, renal insufficiency, respiratory insufficiency, and an episode of delirium.

**Fig 1:**
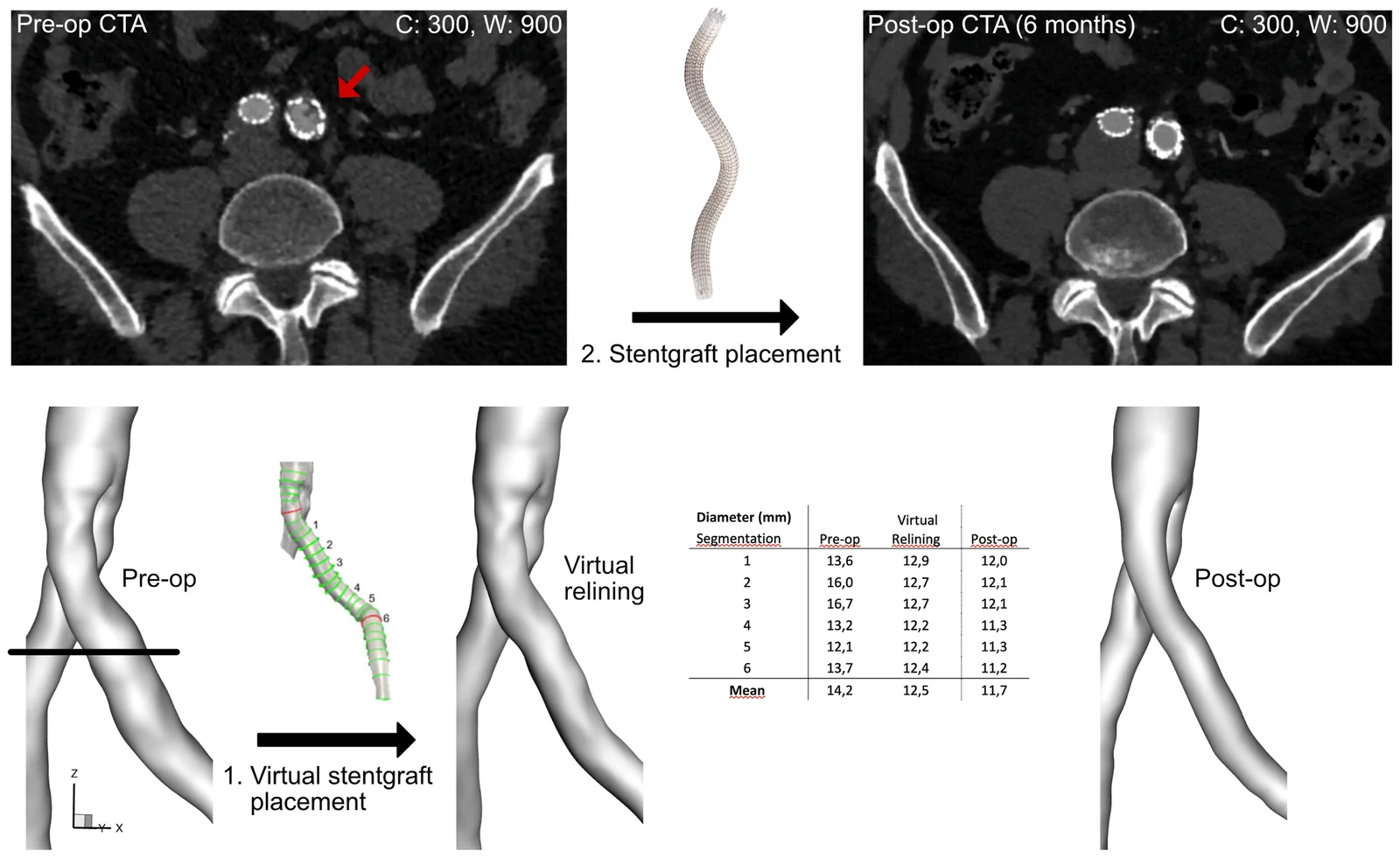
Preoperative and postoperative computed tomography angiography (*CTA*) with corresponding computational fluid dynamics (CFD) analyses. (1) Preoperative CFD model with two geometric models: a segmentation of the preoperative situation, and a geometry where this segmentation was virtually adapted to simulate virtual relining with a VIABAHN stent graft. (2) The postoperative CTA of August 2024 after the actual VIABAHN stent graft placement, with corresponding postoperative CFD model. The table shows differences (mm) in segmentation diameter for slices 1 to 6 across the three models. The preoperative CTA shows an area of intraprosthetic thrombus (*red arrow*) in the stent graft. The location of the axial CTA slice is shown in the preoperative model with a *black interrupted line*.

In 2017, kinking of the left EVAR limb was observed. A relining procedure was performed using a 16-mm Endurant limb (total length, 156 mm; proximal diameter, 16 mm; distal diameter, 13 mm; Medtronic), with a proximal landing zone positioned in the 13-mm distal segment of the Excluder main body and a distal landing zone in the external iliac artery, thus overstenting the left internal iliac artery (Table). In 2022 and 2023, distal thrombotic occlusions were identified, starting from the left femoropopliteal artery extending into the infrapopliteal arteries. Both events were successfully treated with catheter-directed chemical thrombolysis. A cardiac source of thromboembolism was not found and an intraluminal prosthetic thrombus (IPT) in the left iliac limb was therefore considered the most likely source of embolization. Following the intervention in 2022, anticoagulation with apixaban was initiated. In 2023, dual therapy was initiated with the addition of clopidogrel. A follow-up CTA performed in February 2024 demonstrated progressive IPT formation within the left EVAR limb (Fig 1). There were no signs of stenosis or kinking of the endograft. Anticoagulation was switched from apixaban to acenocoumarol. Normal duplex waveforms were observed within the proximal and distal stent graft of the left leg, with peak systolic velocities of 61 and 62 cm/s, respectively.

**Fig 2:**
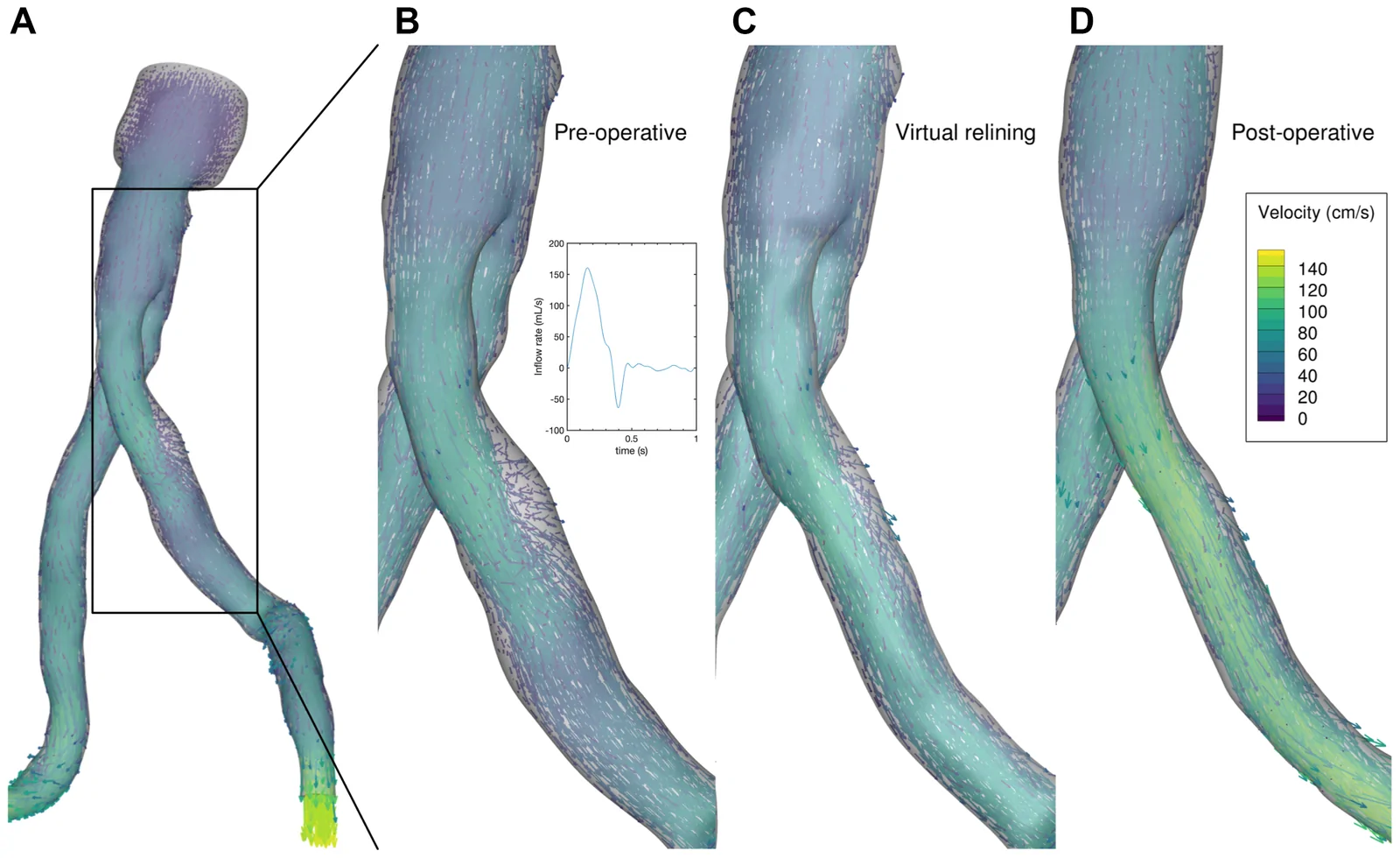
Computational fluid dynamics (CFD) results of the flow velocity (cm/s) in the aortoiliac stent graft, for the preoperative model **(A,B)**, the virtual relining model **(C)** and the postoperative model **(D)**, with the inlet velocity profile derived from duplex ultrasound study **(B)**. The *black square* shows the area of interest. An animated version of velocity over the cardiac cycle is available as Supplementary Video 1 (online only).

**Fig 3:**
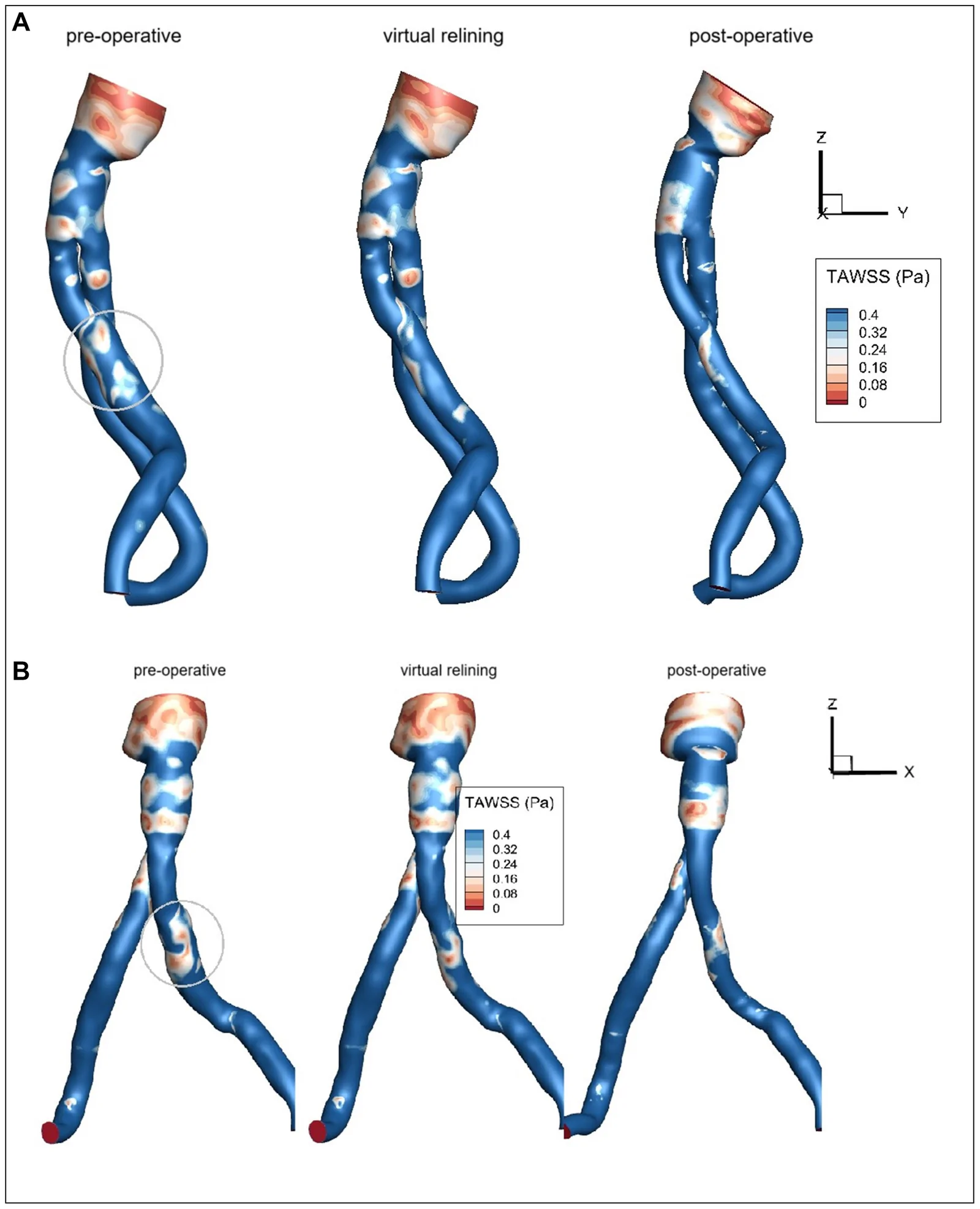
Time-averaged wall shear stress (*TAWSS*, Pa) in the aortoiliac stent graft for the preoperative model (left), the virtual relining model (middle) and the postoperative model (right), from different angles. The *gray circle* shows the area of interest with a low TAWSS (<0.1 Pa).

To investigate hemodynamic causes, a CFD simulation was performed using the SimVascular finite-element solver^10^ (Stanford & UC Berkeley) to search for abnormal blood flow patterns in EVAR limb that might explain the progressive IPT. Segmentation was based on a recent CTA scan with 0.7-mm slice thickness (Fig 1). The segmentation included the full endograft lumen, including the thrombus as flow lumen in the geometry, which assumed that the stent graft geometry was identical before the formation of IPT. The IPT was excluded in the CFD geometry to enable an evaluation of the original stent graft placement on the risk of thrombus formation. The flow rate was derived from a duplex ultrasound (DUS) study and the artery's diameter, under the assumption of Womersley flow profile, and was set as pulsatile inflow condition with a Womersley profile, as previously validated.^11^ The flow distribution over left and right iliac outlets was based on the mean flow split in DUS study and set with Windkessel models.^11^ Further assumptions included a rigid wall and a Newtonian viscosity model.^11^ The simulation showed normal flow in the main body and right limb, whereas the left limb showed abnormal flow with a pronounced area of flow separation and recirculation at the lateral location, where the graft diameter was 16 mm. This region was located distal to a reverse tapered segment, where the diameter increased from 12 to 16 mm, and correlated with the mural thrombus observed on the CTA (Fig 2; Supplementary Video 1, online only). This area was characterized by a streak of TAWSS below 0.1 Pa, with minimum values below 0.04 Pa (Fig 3).

**Fig 4:**
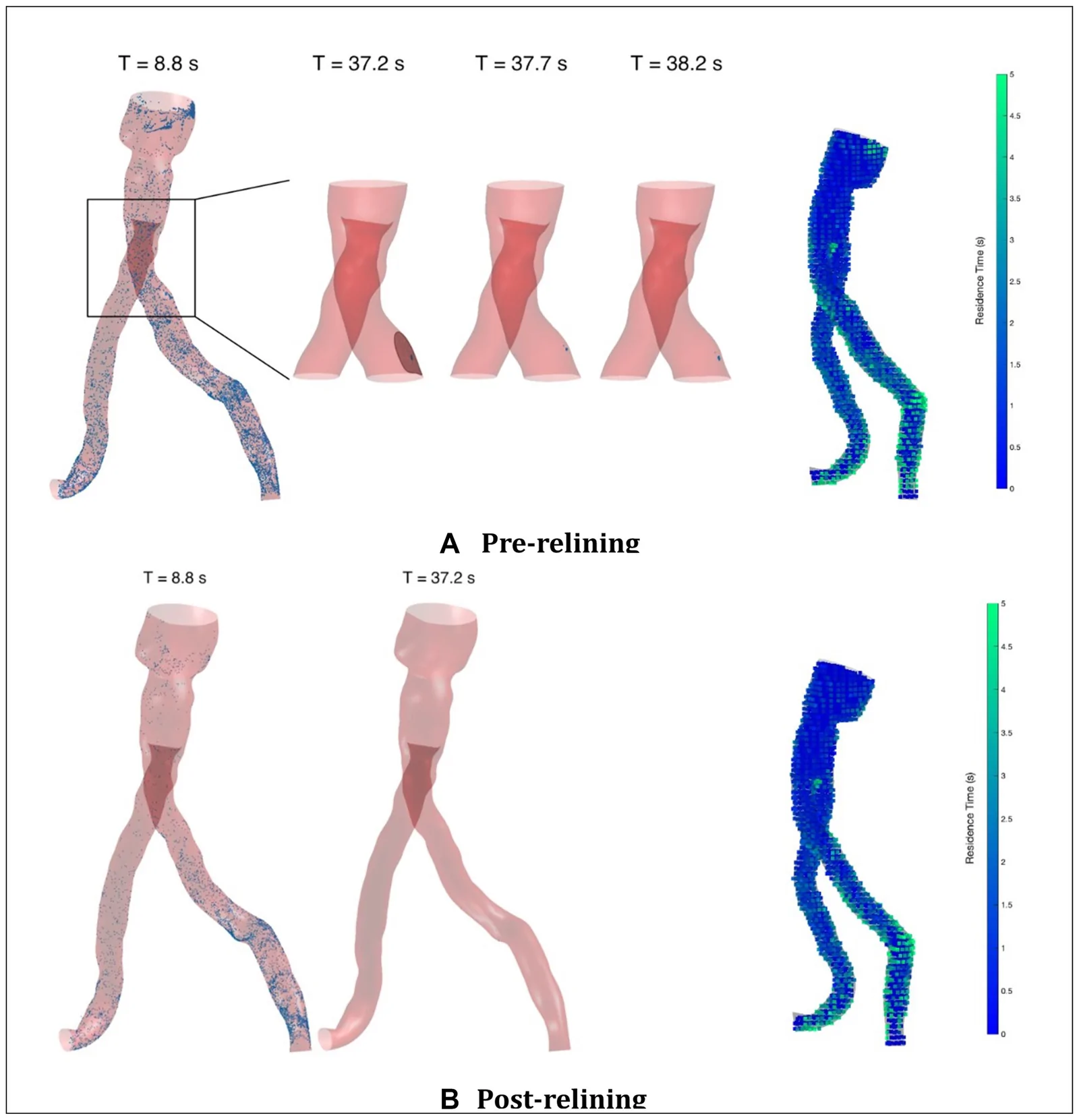
Visualization of the traced particles and residence time **(A)** before and **(B)** after stent graft relining. In **A**, the insert shows a group of about 450 particles that remain circulating in a lateral area of the iliac stent graft. After relining **(B)**, this area with long recirculation time was resolved.

A particle-tracking simulation^12^ to evaluate the RT revealed that a group of particles got entrained within this area of flow recirculation^13^ (Fig 4). In this area, a group of 450 particles (0.16% of released particles) was stuck in a periodic motion spanning 4.4 mm and did not exit the area after 40 seconds of simulation. In other words, they had a RT well over 40 seconds, which is very high even compared with aneurysm RTs.

With the hypothesis that stent relining of the limb with a 13-mm endoprosthesis would eliminate the reverse taper, from 12 to 16 mm at the point of flow separation, a second simulation was performed that mimicked the impact of relining with a 13 × 100 mm VIABAHN endoprosthesis (W. L. Gore and Associates). This option was considered as a reverse tapering cylinder (backwards-facing step) creates a larger region of recirculation that in turn may lead to flow stagnation and thus a more streamlined transition might decrease the risk on thrombus deposits.^4^ The steps for obtaining the virtual geometry of the VIABAHN are detailed in Technical supplement (online only). The CFD simulation of the virtual relining showed a clear improvement in hemodynamics (Fig 2, Fig 3, Fig 4). The area of adverse flow during systole was reduced with an associated 2.3 times reduction in total area of low WSS (<0.1 Pa). For the virtually relined configuration, there was no longer a group of entrained particles in the left limb and RT values normalized to maximum values of 5 seconds.

After a multidisciplinary consultation, and patient informed consent, it was decided to perform the procedure as was simulated. A 13 × 100 mm VIABAHN endoprosthesis was inserted percutaneously, and the postprocedural course was uneventful. The postprocedural CTA confirmed a good positioning of the endograft without signs of thrombus formation. Given the absence of mural thrombus, anticoagulation with acenocoumarol was discontinued and replaced with dual antiplatelet therapy consisting of carbasalate calcium and clopidogrel. One-year after the procedure, no new thromboembolic events did occur, and the CTA showed no thrombus formation inside the endograft, consistent with the CTA performed after the procedure.

**Fig 5:**
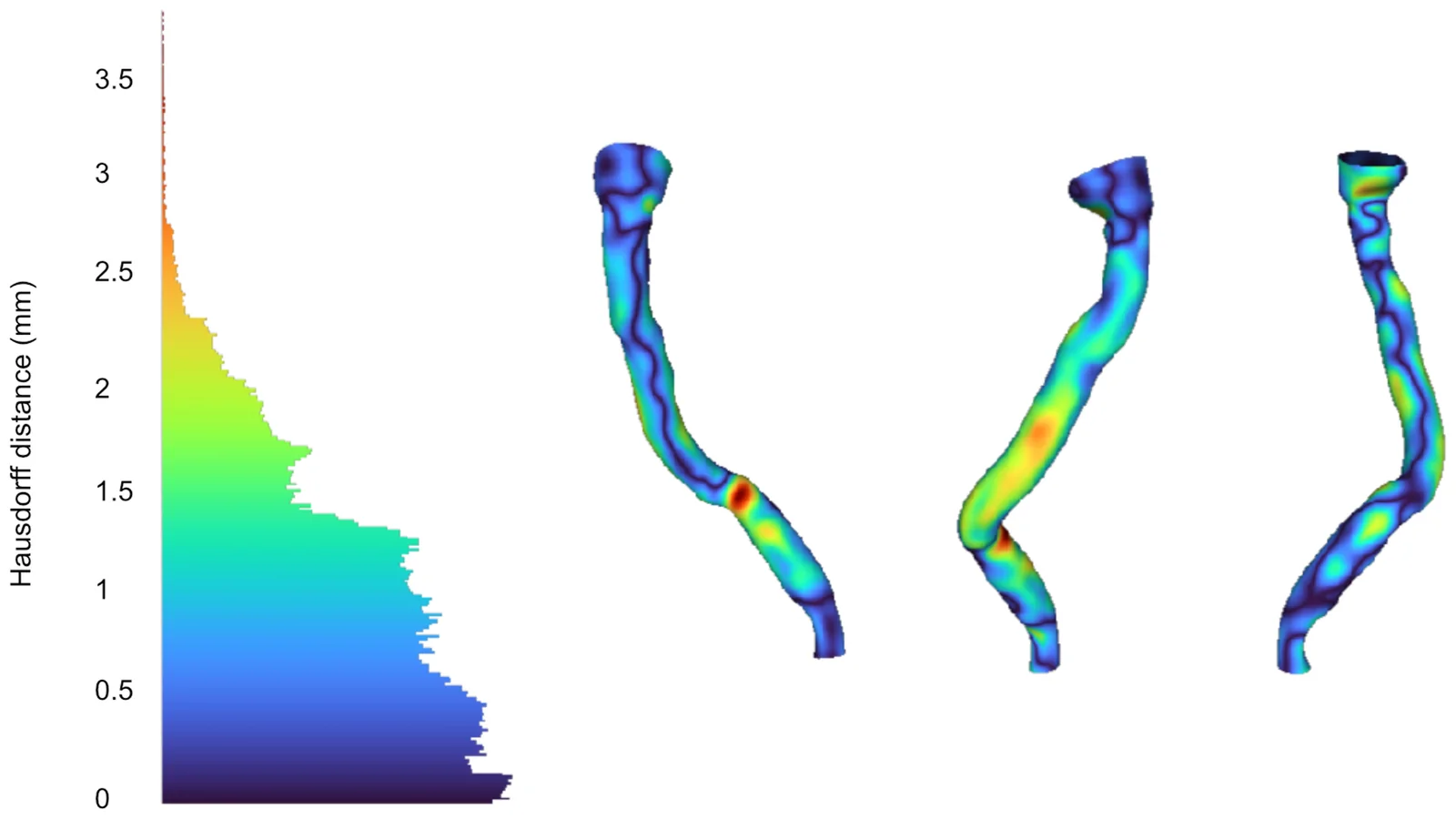
Visualization of the Hausdorff distance between the virtually relined geometry and the post-operative geometry. *Red* indicates maximal outliers and *dark blue* indicates minimal outliers. The geometry is shown from different angles, presented on the virtual relining model.

A geometric comparison showed a mean Hausdorff distance between virtually stented and the postprocedural CTA model of 0.23 mm (range, 0.00-3.73 mm) (Fig 5). More information about the Hausdorff distance can be found in the Technical supplement (online only). A geometric comparison based on segmentation areas of the relined track in Simvascular also confirmed a decrease in diameter, which was even larger than predicted (Fig 1).

A complementary CFD analysis, using the geometry of the postprocedural CTA, demonstrated a reduction of the area with low TAWSS (<0.1 Pa) of 544% compared with the preprocedural geometry. Furthermore, a decrease in the zone of recirculation and an increase in flow velocity in the left limb for the virtually relined and postoperative models was seen, which is in line with mass conservation. For RT analysis of the postoperative configurations, values and observations were similar as the predicted virtually relined geometry.

Informed consent for publication of this case report was obtained from the patient.

## Discussion

In this patient, undergoing EVAR complicated by intraprosthetic mural thrombus formation in the iliac limb and recurrent ipsilateral thromboembolic events, CFD analysis identified regions of pronounced blood flow stasis corresponding to the location of the thrombus. This site was located at an area of reversed tapering in a curved trajectory, a combination that likely contributed to the adverse hemodynamics. In the three-dimensional anatomic and CFD model of the patient, a virtual relining with a smaller diameter stent graft demonstrated that this would improve the hemodynamic environment, with a substantial reduction in flow stasis at the intraprosthetic mural thrombus site. With all other known etiologies, including kinking, stenosis, and coagulation disorders, of limb graft thrombosis ruled out, the relining procedure was performed. The postoperative stent graft configuration and CFD hemodynamics showed a strong match to the predicted situation, and the patient was free of intraprosthetic thrombus and thromboembolic events at 12 months.

From the CFD hemodynamic parameters, WSS and RT were used to quantify areas of flow stasis. We hypothesized that a combination of blood flow stasis and graft material leads to IPT formation. At the site of mural thrombus, the CFD analysis demonstrated recirculating flow, WSS values below 0.04 Pa and a quasi-infinite RT, which was reduced to 5 seconds after relining. We used a Lagrangian method of RT analysis, which is harder to quantify than Eulerian methods but provides more intuitive results.^14^ Previous studies reported associations between WSS and RT and thrombotic events in fenestrated EVAR,^8^ femoral artery stent grafts,^9^ in vitro thrombus formation,^4^ and intraluminal thrombus in intracranial aneurysms.^13^ Although evidence is growing, threshold values for clinical predictions have not been robustly demonstrated and if a single threshold value can be used for various arterial sites and pathologies is unclear. This highlights the need for larger cohort studies to define threshold values.

Luminal thrombus formation is the second most common adverse finding after EVAR with a prevalence up to 20%, usually seen within 1-year.^1^^,^^15^ Risk factors for IPT that have been identified include polyester graft material, iliac limb flare compression, and aortoiliac unibody grafts, associated with significant diameter transitions.^1^^,^^2^^,^^16^ Although common, a meta-analysis did not find a significant association between IPT and thromboembolic events.^1^ Although not reported in this meta-analysis, the majority of included IPT (70%) was present in the main EVAR body.^17^ IPT in an EVAR limb might therefore be associated with thromboembolic events,^16^^,^^18^ as the higher velocities and shearing forces in the limb can more easily dislodge thrombus or activate platelets compared with main body IPT. This hypothesis, which would explain the recurring thrombotic events distal to the EVAR in our studied case, should be tested in future studies.

Although limited to a single patient case, this report underlines the potential of CFD combined with virtual stenting in the management of stent graft thrombosis. If areas of adverse hemodynamics are detected by CFD, relining procedures of various stent diameters or lengths can be easily evaluated in the digital model of the patient. We applied a simplified virtual stenting that assumed that the relined configuration would follow the same centerline as the old configuration. This proved sufficient for estimating the geometric and hemodynamic effect of the relining with a 7.28% overestimation in geometry from the postoperative results. Advanced stent graft deployment simulations^19^ can improve fidelity for very stiff stent grafts or complex configurations, but we demonstrated that for a relining procedure with a relatively flexible stent graft this was not required. If patients at risk of limb graft thrombosis could be identified before the EVAR procedure, virtual treatment of various devices with corresponding CFD assessment can be pursued to find a stent configuration that minimizes thrombus risk. This would require a more intricate simulation of the stent graft deployment, using estimates of the stent graft's and aorta's mechanical properties. Such a hemodynamics-driven approach might be beneficial for patient anatomies that require reverse tapering due to large iliac arteries, especially with concomitant tortuous iliac anatomy.

### Limitations

Next to the hemodynamic environment, the insertion of the heparin-bonded expanded polytetrafluoroethylene (ePTFE) VIABAHN stent graft altered the local biochemical environment in the affected area. As ePTFE grafts are associated with a decreased incidence of IPT compared with in situ polyester graft,^1^ this could have decreased the likelihood of thrombus formation as well. Although low WSS has also been associated with thrombosis in ePTFE grafts,^9^ our study cannot separate the effects of the altered hemodynamic and graft environment on IPT. Clotting and platelet activity is another metric in the thrombosis triad that we did not explicitly measure upon reducing the level of antithrombotic medication during follow-up, but this would have increased rather than decreased the likelihood of thrombus formation.

Uncertainties in input data and modeling simplifications could affect the outcome of the CFD simulations. It is known that the rigid wall assumption^20^ and Newtonian rheology model^21^ have only small effects on time-averaged metrics such as TAWSS. The CFD outcome was likely more sensitive to potential inaccuracies in the geometric reconstruction from CTA and the inflow boundary condition from DUS study.^11^ However, we deem the effects of geometric inaccuracies small relative to the reverse tapering of 3 to 4 mm. This was further supported by the similarity of geometric and hemodynamic findings in the virtually simulated relining procedure and the postoperative scan.

Although this study reports a single, complex case, evaluation in larger series with different limb geometries with and without thrombus formation could substantiate the suggested relation between hemodynamics and thrombotic risk. The value of CFD is greatest when hemodynamic parameters can be linked to early, localized IPT on CTA, which serves as an important intermediate stage preceding complete limb graft occlusion and enables identification of thrombus initiation sites. However, such intermediate imaging before full limb graft occlusion occurs is rarely available. For our CFD-analysis, a CTA-scan with IPT was used to evaluate the configuration before IPT was present, in order to evaluate thrombus formation risk. A high-resolution CTA with thin slices of the configuration after IPT formation was available, but not before IPT. The CTA with IPT was therefore used, which assumed that the stent graft geometry was not altered during the formation of IPT. Future work could focus on CFD analysis of repeated CTA scans of thrombus initiation and progression, and augment the CFD with a model of thrombus growth and remodeling.

## Conclusions

This case report demonstrated how CFD modeling may help identify hemodynamic conditions associated with EVAR limb thrombus formation and may assist in guiding stent graft relining options to improve the local hemodynamic environment.

## Funding

No funding was provided.

## Disclosures

M.R. has received research funding and speaker fees from Abbott Vascular, W.L. Gore and associates and Medtronic. L.V. has received research funding from Abbott Vascular and Medtronic. The remaining authors (L.S. and P.S.) have no conflict of interest.

## Appendix (online only)

Technical Supplement

Video 1 Computational fluid dynamics (CFD) results of the flow velocity (cm/s) in the aortoiliac stent graft, for the preoperative model, the predicted postoperative model after virtual relining and the postoperative model after relining.
