# Supplementary material for: Using patient-specific computational fluid dynamics to assess intraprosthetic thrombus formation risk and guide relining after endovascular aneurysm repair
Source: JVS Vasc Sci. 2026 Aug 5;7:100437. doi: 10.1016/j.jvssci.2026.100437 (PMC13572162; doi:10.1016/j.jvssci.2026.100437)
Supplement: Supplementary file 1 [file mmc1.docx]

# Technical supplement

## Geometric reconstruction and mesh generation

Geometric reconstruction and mesh generation were performed in Simvascular^1^. First, a 3D model was made based on patient's CTA (0.7-mm slice thickness), by segmenting the arterial lumen of interest using pathlines, 2D-contour segmentations employing level-set algorithms with spline-based shape fitting and manual adjustments for stent artifacts*.*

Mesh convergence analysis was performed, with convergence defined as a TAWSS change < 5 % with mesh size doubling. The meshes used for analysis had 1.59 – 1.65 million cells^2^.

For the reconstruction of the stentgraft prediction, the 2D segmentations at the level of the 16-mm Excluder stent graft, were proportionally decreased in area to match the area of a13-mm stent graft. . The shape of the segmentation contours remained the same.

## Boundary conditions

The flow distribution over left and right iliac outlets was based on the mean flow split in DUS, where an approximately equal flow rate split was observed, and set with Windkessel models. For the outlet flows on DUS, this equal flow rate split resulted , in RCR boundary conditions with resistances of 8.76E-5 and 7.88E-4 (kg · mm^-4^ · s^-1^ , proximal and distal resistance) and a capacitance of 2.28E3 mm^4^ · s^2^ · kg^-1^ for both outlets.

## Assumptions and material properties

Assumptions of rigid wall^4^ and Newtonian rheology model^5^ were made. For this case, blood viscosity is set at 4 · 10^-6^ kg/mm.s with a fluid density of 1.06 ·10^-6^ kg/mm^3^. Lastly, the simulation is performed for 400 timesteps, with a time step size of 1 · 10^-3^ s, simulating two cardiac cycles of which the last one is used for analysis.

## Data analysis

### Hemodynamic parameters

From the CFD simulation, the time-averaged wall shear stress (TAWSS) and the residence time (RT) were analysed. Visualisations of flow velocity and TAWSS and flow velocity were performed with the use of Tecplot EX 360 2024 (Tecplot, Inc., Bellevue, WA, USA).

**Residence time**

The pulsatile CFD velocity field was used as input for the residence time simulation. This velocity field was modified with an inward-pointing velocity of 0.5 mm/s added to the wall points to prevent particles from leaving the fluid domain through the wall ^6^.

For the residence time simulation, particles were seeded throughout the model at the CFD mesh points (1 particle per mesh point excluding wall points, yielding approximately 280.000 particles for the particle tracking simulation) and released at timepoint 0. The particles were tracked in a Lagrangian fashion with an in-house simulation algorithm (Matlab 2022b, MathWorks, Natick, MA), using linear interpolation to query velocity values between mesh points and a 2-ms time step for updating particle positions. All particles were tracked for 40 seconds or until they left the computational domain through the inlet or outlet.

Residence time (RT) was computed for 5×5×5 mm3 sections of the aneurysm, defined as the time elapsed for the particle concentration to fall under 2% of the initial seeding concentration^7^.

### Hausdorff distance

The average Hausdorff distance is a performance measurement that calculates the distance between two point sets, as seen in equation 1 for two finite point sets X and Y^8^. So for each point on the generated mesh, it calculates the distance to the closest point on the predicted mesh. MeshLab 2023.12 software is used to calculate the average Hausdorff distance between the predicted Viabahn model and the post-operative model^9^. The choice is made to only compare the area of the Viabahn placement and not the entire model, as this was easier for alignment. The caps of both models are removed and both models are remeshed with a factor 0.2. The models needed translation and rotation first, so alignment is correct. In this case, first a translation of the post-operative mesh of [-8, 128, -587] and for the predicted Viabahn model of [-8, -128, 586] had to be made, for the initial stent relining. Second, a translation of [-18.6, -33.6, 0.9] with a rotation of [2, -0.8, -6] on the predicted Viabahn mesh was performed. For a complete overview and to make sure the optimal translation is chosen, a deviation of -0.1 and +0.1 is taken into account for each of the translation directions and a deviation of –0.01 and +0.01 for each rotation direction (Table S1). The optimal coordinates are chosen by the alignment with the lowest mean value and corresponding low RMS.

1)

Table S1: Optimum Hausdorff Distance translation (X*_t_*, Y*_t_*, Z*_t_* ) and rotation (X*_r_*, Y*_r_*, Z*_r)_*. To see if the optimum translation and rotation for the Hausdorff Distance is applied, a deviation of +- 0.1 is tested for the X,Y,Z-translation and a deviation of +- 0.01 is tested for the X,Y,Z-rotation. The primary outcome is to find the lowest mean value. For the optimum and all deviations, the minimum is equal to 0.0. Within the deviations, there is a maximal change in mean Hausdorff Distance of 0.11 mm (48.8 %).

|  | **Adaption** | **Max** | **Mean** | **RMS** |
| --- | --- | --- | --- | --- |
| **Optimum** | [-18.6, -33.6, 0.9, 2, -0.8, -6]  [X*_t_*, Y*_t_*, Z*_t_*, X*_r_*, Y*_r_*, Z*_r_*] | 3.728 | 0.226 | 0.546 |
| **X-translation** | -0.1 | 3.766 | 0.227 | 0.547 |
|  | +0.1 | 3.703 | 0.286 | 0.533 |
| **X-Rotation** | -0.01 | 3.857 | 0.266 | 0.553 |
|  | +0.01 | 3.599 | 0.264 | 0.547 |
| **Y-translation** | -0.1 | 3.817 | 0.255 | 0.550 |
|  | +0.1 | 3.639 | 0.253 | 0.546 |
| **Y-Rotation** | -0.01 | 3.688 | 0.308 | 0.529 |
|  | +0.01 | 3.768 | 0.336 | 0.588 |
| **Z-translation** | -0.1 | 3.690 | 0.226 | 0.545 |
|  | +0.1 | 3.766 | 0.227 | 0.547 |
| **Z-Rotation** | -0.01 | 3.721 | 0.234 | 0.544 |
|  | +0.01 | 3.735 | 0.236 | 0.548 |

## References

1.	Updegrove A, Wilson NM, Merkow J, Lan H, Marsden AL, Shadden SC. SimVascular: An Open Source Pipeline for Cardiovascular Simulation. *Annals of Biomedical Engineering 2016 45:3*. 2016;45(3):525-541. doi:10.1007/s10439-016-1762-8

2.	Velde L van de, Helvert M van, Engelhard S, Ghanbarzadeh-Dagheyan A, Mirgolbabaee H, Voorneveld J et al. Validation of ultrasound velocimetry and computational fluid dynamics for flow assessment in femoral artery stenotic disease. doi:10.1117/1.JMI.11.3.037001

3.	McGah PM, Nerva JD, Morton RP, Barbour MC, Levitt MR, Mourad PD et al. In vitro validation of endovascular Doppler-derived flow rates in models of the cerebral circulation. *Physiol Meas*. 2015;36(11). doi:10.1088/0967-3334/36/11/2301

4.	Boccadifuoco A, Mariotti A, Capellini K, Celi S, Salvetti MV. Validation of Numerical Simulations of Thoracic Aorta Hemodynamics: Comparison with In Vivo Measurements and Stochastic Sensitivity Analysis. *Cardiovasc Eng Technol*. 2018;9(4):688-706. doi:10.1007/S13239-018-00387-X/METRICS

5.	Tzirakis K, Kontopodis N, Ioannou C V. A hemodynamic study of blood flow models on various stent graft configurations during aorto-iliac reconstruction. *Clin Hemorheol Microcirc*. 2024;87(2):199-219. doi:10.3233/CH-232058,

6.	Di Achille P, Tellides G, Figueroa CA, Humphrey JD. A haemodynamic predictor of intraluminal thrombus formation in abdominal aortic aneurysms. *Proceedings of the Royal Society A: Mathematical, Physical and Engineering Sciences*. 2014;470(2172). doi:10.1098/rspa.2014.0163

7.	Rayz VL, Boussel L, Ge L, Leach JR, Martin AJ, Lawton MT, et al. Flow residence time and regions of intraluminal thrombus deposition in intracranial aneurysms. *Ann Biomed Eng*. 2010;38(10):3058-3069. doi:10.1007/s10439-010-0065-8

8.	Aydin OU, Taha AA, Hilbert A, Khalil AA, Galinovic I, Fiebach FB et al. On the usage of average Hausdorff distance for segmentation performance assessment: hidden error when used for ranking. *Eur Radiol Exp*. 2021;5(1). doi:10.1186/S41747-020-00200-2

9.	Cignoni P, Callieri M, Corsini M, Dellepiane M, Ganovelli F, Ranzuglia G. MeshLab: an Open-Source Mesh Processing Tool. In: *Sixth Eurographics Italian Chapter Conference*. 2008:129-136.
